# Explainability of deep neural networks for MRI analysis of brain tumors

**DOI:** 10.1007/s11548-022-02619-x

**Published:** 2022-04-23

**Authors:** Ramy A. Zeineldin, Mohamed E. Karar, Ziad Elshaer, ·Jan Coburger, Christian R. Wirtz, Oliver Burgert, Franziska Mathis-Ullrich

**Affiliations:** 1 Institute for Anthropomatics and Robotics, Karlsruhe Institute of Technology (KIT), 76131 Karlsruhe, Germany; 2 Research Group Computer Assisted Medicine (CaMed), Reutlingen University, 72762 Reutlingen, Germany; 3 Faculty of Electronic Engineering (FEE), Menoufia University, Menouf, 32952 Egypt; 4 Department of Neurosurgery, University of Ulm, 89312 Günzburg, Germany

**Keywords:** Brain glioma, Computer-aided diagnosis, Convolutional neural networks, Explainable AI

## Abstract

**Purpose:**

Artificial intelligence (AI), in particular deep neural networks, has achieved remarkable results for medical image analysis in several applications. Yet the lack of explainability of deep neural models is considered the principal restriction before applying these methods in clinical practice.

**Methods:**

In this study, we propose a NeuroXAI framework for explainable AI of deep learning networks to increase the trust of medical experts. NeuroXAI implements seven state-of-the-art explanation methods providing visualization maps to help make deep learning models transparent.

**Results:**

NeuroXAI has been applied to two applications of the most widely investigated problems in brain imaging analysis, i.e., image classification and segmentation using magnetic resonance (MR) modality. Visual attention maps of multiple XAI methods have been generated and compared for both applications. Another experiment demonstrated that NeuroXAI can provide information flow visualization on internal layers of a segmentation CNN.

**Conclusion:**

Due to its open architecture, ease of implementation, and scalability to new XAI methods, NeuroXAI could be utilized to assist radiologists and medical professionals in the detection and diagnosis of brain tumors in the clinical routine of cancer patients. The code of NeuroXAI is publicly accessible at https://github.com/razeineldin/NeuroXAI.

## Introduction

Brain and other nervous system tumors (ONS), including the glioblastoma (GBM), are among the leading cause of cancer death in adults [1, 2]. Brain cancer, explicitly malignant and benign, represents the second major source of cancer-related deaths in young adults and children [1]. Common treatment options for brain cancer include surgical intervention, radiotherapy, and chemotherapy [3]. Nevertheless, physically localizing and resecting pathological targets by surgery is almost impossible, owing to the difficulty in visually distinguishing brain tumors from surrounding brain parenchyma [4].

In practice, magnetic resonance imaging (MRI) can help physicians detect brain tumors by providing soft tissue imaging allowing improved tumor localization and boundary definition [5]. By varying the weightage of image contrast, the anatomy of the human brain, blood–brain barrier, and brain tumor boundaries could be detected and visualized. Multi-parametric MRI includes native T1-weighted (T1W), post-contrast T1-weighted (T1Gd), T2-weighted (T2W), and T2 fluid-attenuated inversion recovery (FLAIR). However, interpreting these multi-modal images can be highly challenging for physicians to analyze and provide diagnosis, make intraoperative decisions in a short time as wrong remedy procedures could lead to patient discomfort physically and financially [3].

Computer-aided diagnosis systems (CADs) aid in these cases to detect brain tumors using multimodal MRI scans, minimizing these inconveniences [6]. CADs are computer systems that assist radiologists and physicians in the interpretation, analysis, and evaluation of MRI data comprehensively in a short time, e.g., brain tumor segmentation and predicting histological grades of intracranial neoplasms [7, 8].

Recent developments in the field of artificial intelligence (AI), especially deep learning (DL), have led to a renewed interest in analyzing brain cancer, its causes, and its various development phases [9, 10]. In medical applications, there are typically fewer data samples with higher complexity compared with other applications. Among the numerous segmentation techniques, convolutional neural networks (CNN) have attracted much attention for medical image understanding tasks like image classification or multimodal tumor segmentation. For instance, U-Net variants [11, 12], which use symmetric encoder–decoder architecture, have performed state-of-the-art results for medical image segmentation. Similarly, several publications have appeared in recent years for accurate medical classification including [8, 13–16]. Hence, employing DL technologies in CADs could potentially expand physicians’ capabilities assisting in perioperative evaluation of intracranial pathologies and enhancing the efficiency of postoperative follow-up [9, 10].

Nevertheless, the introduction of DL techniques in the clinical environment is still limited due to some restrictions [17]. The most significant one is that DL strategies consider only the input images and the output results, without any transparency of the underlying information flow in the network internal layers. In sensitive applications such as brain imaging applications, it is crucial to understand the reason behind the network prediction to ensure that the model provides the correct estimation. Accordingly, explainable AI (XAI) has gained a substantial interest to explore the “black box” DL networks in the medical field [17, 18]. XAI methods allow researchers, developers, and end-users to obtain transparent DL models that can describe their decisions to humans in an understandable manner. For medical end-users, the demand for explainability is increasing to create their trust in DL techniques and to encourage them to utilize these systems for assisting the clinical procedures. Moreover, the European Union data protection law, titled General Data Protection Regulation (GDPR), imposes the explanation as a requirement for automated learning systems before being used with patients clinically [19].

## Related work

Generally, XAI techniques in medical imaging can be grouped into perturbation-based or gradient-based approaches. Perturbation-based methods investigate the network by changing the input features and measuring the impact on the output estimations by a forward training of the model. Some examples include LIME [20], SHAP [20], deconvolution [21], and occlusion [21]. Gradient-based XAI methods have been widely adopted to provide feature attribution maps by calculating the partial derivative of the output predictions through every layer of the neural network with respect to (w.r.t) the input images. These techniques have the advantage of being post hoc, meaning that they are applied after the training phase of the DL model avoiding the accuracy vs explainability trade-off. In addition, they are usually fast compared with perturbation approaches since their runtime does not depend on the number of input features. A number of publications have been reported for back-propagating approaches such as Vanilla gradient [22], guided backpropagation [23], integrated gradients [24], guided integrated gradients [25], SmoothGrad [26], Grad-CAM [27], and guided Grad-CAM [27]. Several XAI methods have been previously proposed for natural image tasks, while little attention has been paid to explain brain imaging applications [18]. For brain cancer classification, Windisch et al. [28] applied 2D Grad-CAM to generate heatmaps indicating which areas of the input MRI made the classifier decide on the category of the existence of a brain tumor. Similarly, 2D Grad-CAM was used in [29] to evaluate the performance of three DL models in brain tumor classification. The key limitation of these studies is that experiments were concluded on 2D MRI slices without investigating the model on 3D medical applications.

Explainable learning has been applied as well for brain glioma segmentation [30, 31]. In [30], 2D Grad-CAM was applied to extract explanations for the deep neural networks for brain tumors identification. It suffers from the same limitations associated with the previous classification explanation methods of being 2D only. Another approach was introduced in [31] that extends class activation mapping (CAM) [32] by generating 3D heatmaps to visualize the importance of segmentation output. Despite being highly class-discriminative, it made a trade-off between the model complexity and the performance to make CNNs transparent.

In this paper, our main goal is to develop a new NeuroXAI framework for obtaining 2D and 3D explainable sensitivity maps to assist clinicians to understand and trust the performance of DL algorithms in clinical procedures. Hence, the contribution of this study has threefold:

1. A new explainability framework, namely NeuroXAI, is proposed to make the current DL models for brain imaging research interpretable without any architecture modification or performance degradation.
2. NeuroXAI included seven state-of-the-art backpropagating XAI techniques for generating 2D and 3D visual interpretations of CNN output.
3. A comprehensive evaluation of the proposed framework demonstrated promising explanation results for two showcases of MRI classification and segmentation of brain tumors.

## Methods

### NeuroXAI

**Fig. 1:**
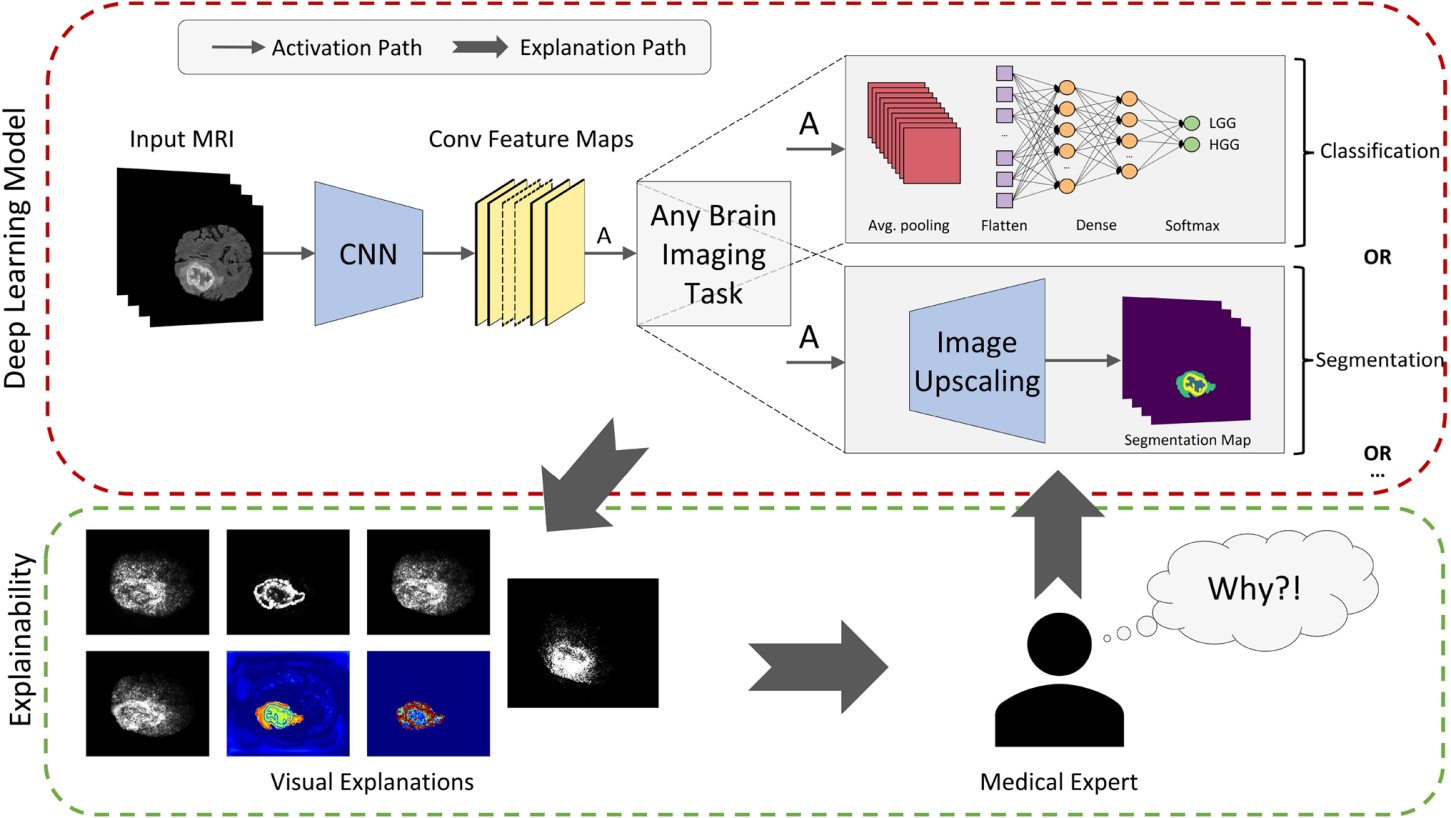
Pipeline of the proposed NeuroXAI framework

The overall pipeline of NeuroXAI is shown in Fig. 1. It consists of two main parts, which are a deep neural network to achieve processing tasks of the brain images and an explanation generator. Given brain MRI volumes as input, the images are forward propagated through the CNN generating convolutional feature maps and then through task-specific computations to obtain the desired output (e.g., category prediction in case of classification and/or tumor segmentation). Afterward, the network output is presented to medical professionals to assess the findings and request an explanation if necessary. Finally, visual explanation maps are provided by the explainability part to interpret the results of applied deep neural networks. This can be achieved using state-of-the-art XAI methods.

While the utilized explanation methods were primarily proposed for interpreting deep image classification, our proposed framework provides an adaption approach to medical image segmentation as well. Further, NeuroXAI converts the segmentation task into a multi-label classification task. This is achieved through global average pooling for each class on the output prediction layer. Therefore, our NeuroXAI offers state-of-the-art XAI methods for classification and segmentation for both 2D and 3D medical image data.

### Vanilla gradient

Vanilla gradient (VG) [22] is the simplest form of visualizing regions of the image that contributes most to the classification output of the neural network. This computes the saliency map by making a single backward pass of the activation of the output class after a forward pass over the network, which can be defined as computing the VG of the output activation w.r.t the input image. Let *P*_*c*_*(Im)* be the prediction of class *c*, computed by the classification layer of the CNN for an input image *X*^*I*^. The objective of Vanilla gradient is to find the L_2_-regularized image, which has the maximum *P*_*c*_, while $\uplambda$ is the regularization term:

1 $$VG={argmax}_{c}{P}_{c}\left({X}^{I}\right)-\lambda {\Vert {X}^{I}\Vert }_{2}^{2}$$

### Guided backpropagation

An alternative way of calculating the gradient of a particular output w.r.t the input is by using guided backpropagation (GBP) [23]. The GBP is a new variant of the deconvolution approach [21] for visualizing the region of interest of an image that most activates a given class. Suppose *F* be the output of a convolutional layer *l* from *L* layers in a multi-layer CNN, and *B* denotes the resultant image from backpropagation:

2 $${B}_{i}^{l}=\left({F}_{i}^{l}>0\right).\left({B}_{i}^{l+1}>0\right).{B}_{i}^{l+1}$$

3 $${B}_{i}^{l+1}=\frac{\partial {F}_{i}^{L}}{\partial {F}_{i}^{l+1}}$$

### Integrated gradients

Sundararajan et al. [24] introduced integrated gradients (IG) to mitigate the saturation problem of gradient-based methods. Let a function *F*: *R*^n^ → [0, 1] denote a deep neural network which has *X*^*I*^ = *γ (α* = *1)* ∈ *R*^*n*^ as the input image, while *X*^*B*^ = *γ (α* = *0)* ∈ *R*^*n*^ represents the baseline. The baseline is simply a black image with all values set to zeros. The IG can be computed by accumulating the gradients at all points on the straight-line path from the baseline *X*^*B*^ to the input image *X*^*I*^:

4 $${IG}_{i}(x)={\int }_{\alpha =0}^{1}\frac{\partial F(\gamma (\alpha ))}{\partial {\gamma }_{i}(\alpha )}\frac{\partial {\gamma }_{i}(\alpha )}{\partial \alpha }d\alpha $$

Here, *i* is the feature for the input image, whereas *α* represents interpolation constant to perturb image features.

### Guided integrated gradients

Kapishnikov et al. [25] proposed guided integrated gradients (GIG) as an adaption of the attribution path based on the input image, baseline, and the deep model to be explained. Similar to IG, the GIG calculates the gradients on the path (*c*) which starts at the baseline (*X*^*B*^) and ends at the input being explained (*X*^*I*^). However, the GIG path (*c*) is determined at every step as opposed to the fixed direction of the IG. This means that GIG finds a subset of features (*S*) that have the least importance among all features toward the input image. Mathematically,

5 $${GIG}_{i}\left({X}^{B},{X}^{I},F\right)=\frac{\partial {\gamma }_{i}^{F}\left(\alpha \right)}{\partial \alpha }=\left\{\begin{array}{c}{x}_{i}^{I}-{x}_{i}^{B}, if i \epsilon S,\\ 0 , otherwise.\end{array}\right.$$

6 $$S={argmin}_{i}\left(Y\right)$$

7 $${y}_{i}=\left\{\begin{array}{c}\left|\frac{\partial F(x)}{\partial {x}_{i}}\right|, if i \epsilon \left\{j|{x}_{j}\ne {x}_{j}^{I}\right\}\\ \infty , otherwise.\end{array}\right.$$

### SmoothGrad

Smilkov et al. [26] presented an improvement for the common problem of gradient-based methods. SmoothGrad [26] solved this problem by providing visually sharpened sensitivity maps. It computes the gradient over multiple samples surrounding the input *X*^*I*^, and the average is calculated after adding Gaussian noise. More formally,

8 $$\overline{{M }_{c}}({X}^{I})=\frac{1}{n}\sum_{1}^{n}{M}_{c}({X}^{I}+\fancyscript{g}(0,{\sigma }^{2}))$$

where ${{M}}_{{c}}({{X}}^{{I}})$ is the original sensitivity map, n is the number of samples, and $\fancyscript{g}(0,{\sigma }^{2})$ denotes Gaussian noise with variance ${\sigma }^{2}$. In general, ${{M}}_{{c}}({{X}}^{{I}})$ can be any gradient-based visualization method, such as explanation methods in the previous sub-sections.

### Grad-CAM

The authors in [27] extended the class activation mapping (CAM) visualization technique to a wide variety of CNNs. The proposed gradient CAM (GCAM) produces visual explanations without re-training or modifications in the model architecture. The gradient of any target class *c* is first computed, and the activation feature map *M* of a specific layer *l* is globally averaged over the width, height, and depth dimensions. Then, the class-discriminative heatmap of GCAM is obtained using a weighted combination of these activation maps, followed by the ReLU function. Here, ${\alpha }_{l}^{c}$ denotes the neuron importance weights.

9 $${GCAM}_{l}^{c}=ReLU(\sum_{l}{\alpha }_{l}^{c}{M}^{l})$$

10 $${\alpha }_{l}^{c}=\frac{1}{N}\sum_{x}\sum_{y}\sum_{z}\frac{\partial {y}^{c}}{\partial {A}_{x,y,z}^{l}}$$

### Guided Grad-CAM

Guided GCAM (GGCAM) was introduced to provide higher-resolution visualizations capturing fine-grained details of the object of interest [27]. GGCAM fuses the point-space gradient visualization method GBP [23] and the class-discriminative coarse heatmaps of GCAM through element-wise multiplication. The estimated saliency map of GCAM is first upsampled to the input *X*^*I*^ spatial resolution using bilinear interpolation before applying the point-wise multiplication with GBP.

## Experiments

### Data

MRI data from the BraTS challenges 2019 and 2021 [33–36] have been used in this study for accomplishing the classification and segmentation tasks. Each subject has four MRI sequences including preoperative multimodal MRI scans of native T1W, Gadolinium T1Gd, T2W, and FLAIR, acquired from multiple different institutions. Although the main aim of the challenge is to compare the best algorithms for segmenting the enhancing tumor (ET), the tumor core (TC), and the whole tumor (WT) regions, the BraTS 2019 dataset also provides classification labels for gliomas. BraTS 2019 database comprises 259 cases of high-grade gliomas (HGG) and 76 cases of low-grade gliomas (LGG), which were used for the first showcase. The second showcase applies the BraTS 2021 database, which contains 1251 MRI images with ground truth annotations without any explicit glioma classification.

Since MRI sequences were acquired using multi-parametric instruments in multi-location centers, input images are needed to be standardized. A preprocessing stage has been applied to all MRI scans, specifically min–max scaling of each MRI modality using z-score normalization, and image cropping to a spatial resolution of 192 × 224 × 160. During the training, data augmentation was applied random flipping, random rotations, intensity transformation, as well as dynamic patch augmentation cropping size of 128 × 128 × 128 to avoid overfitting problems.

### Implementation

**Fig. 2:**
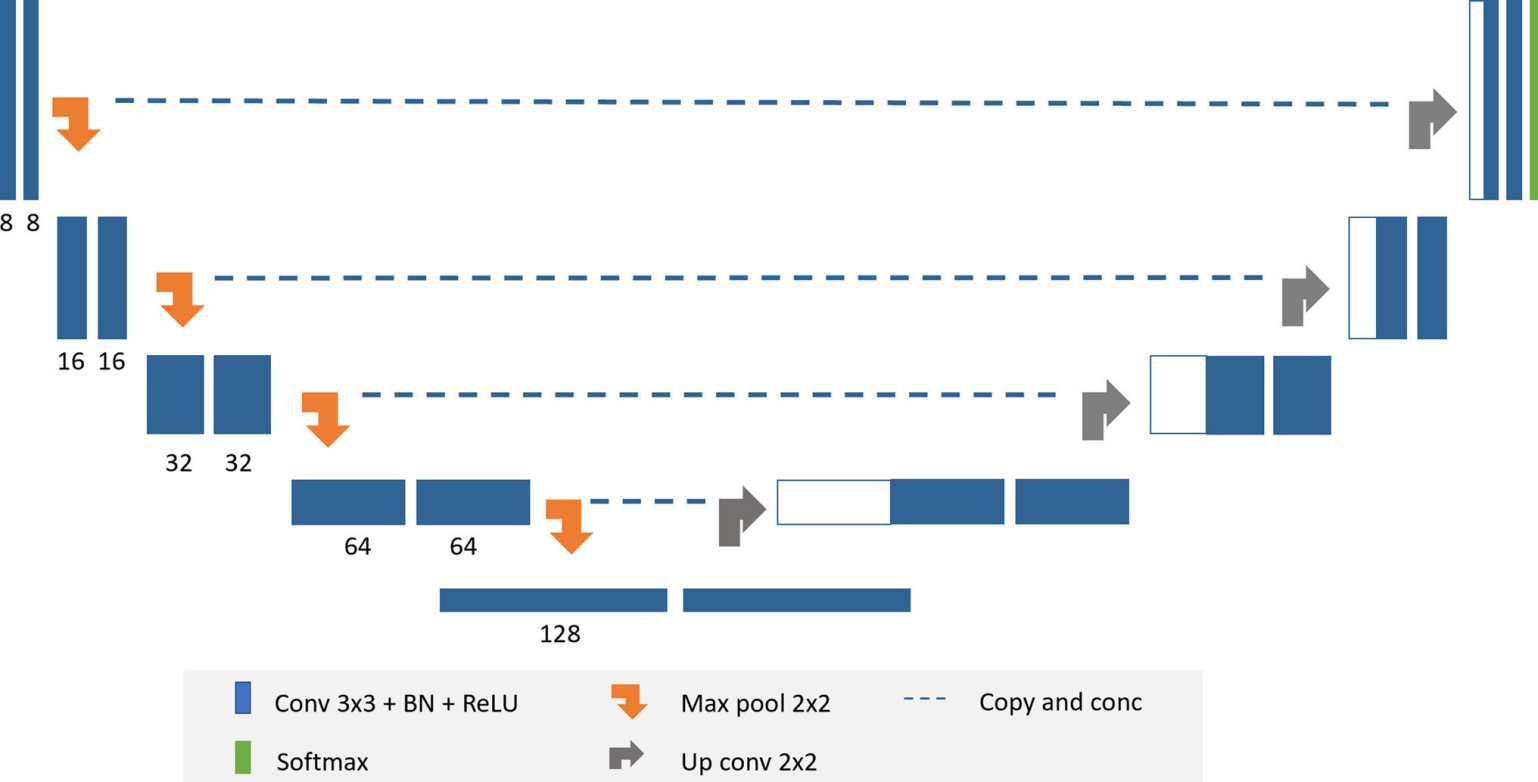
Overview of the architecture details of 3D CNN for glioma segmentation [38]

For the classification task, we employed a simple classifier based on a pretrained ResNet [37] because of its accurate classification results. Deep transfer learning was then adopted to make the model capable of extracting features from brain MR images. Table 1 summarizes the added top layers to the ResNet-50 in our experiment. For the segmentation task, an encoder–decoder neural network was utilized, named 3D DeepSeg [38]. The structure of our network is shown in Fig. 2.

**Table 1:** List of the added top layers to the standard ResNet-50

Type	Output	Feature maps
Average Pooling 2D	2 × 2	512
Flatten	2048	1
Dense	256	1
Dropout	256	1
Dense	2	1

Both DL models were implemented using the TensorFlow library [39] version 2.4. Adam optimizer [40] was used to update the weights of the network, with an initial learning rate of 1e ^– 3^ and 1e ^– 4^ at the very beginning, and the maximum number of training epochs is set to 150 and 1000, and batch size of 64 and 5 for the classification and segmentation networks, respectively. Training the networks was performed on a single NVIDIA graphic card (RTX 2080Ti with 11 GB RAM or RTX 3060 with 12 GB RAM). Explainability experiments were carried out after the training of the original neural network because of using post hoc XAI methods without network re-training or architecture modifications. The final sensitivity maps were generated by our proposed NeuroXAI framework with the pretrained saved weights for both DL models.

## Results

### Showcase I: application to classification

Here, we introduce the application of NeuroXAI to generate visual explanations for automatic brain glioma grading using DL. The main objective of this study is to illustrate the explainability capabilities of our proposed NeuroXAI framework for assisting clinicians, not to obtain the best classification results only. However, the applied classifier achieved a superior accuracy of 98.62%, comparing to the state-of-the-art methods [8, 13–16] as given in Table 2.

**Table 2:** Comparison of our proposed classifier and other deep models in previous studies

Model/ Year	Preprocessing	Method	Accuracy
Ge et al. 2018 [8]	Class balancing and tumor masks	2D CNN	90.87%
Ge et al. 2020 [13]	Tumor mask enhancement	GAN*	88.82%
Mzoughi et al. 2020 [14]	Intensity normalization, contrast enhancement, and cubic B-spline resizing	3D CNN	96.49%
Ahuja et al. 2020 [15]	Data normalization	VGG	99.30%
Dixit and Nanda 2021 [16]	Grayscale conversion and tumor segmentation	IWOA-RBNN**	96%
Our classifier	Z-score normalization, image cropping, and transfer learning	ResNet-50	98.62%

*GAN Generative adversarial network

**IWOA-RBNN Improved whale optimization algorithm for radial basis neural network

**Fig. 3:**
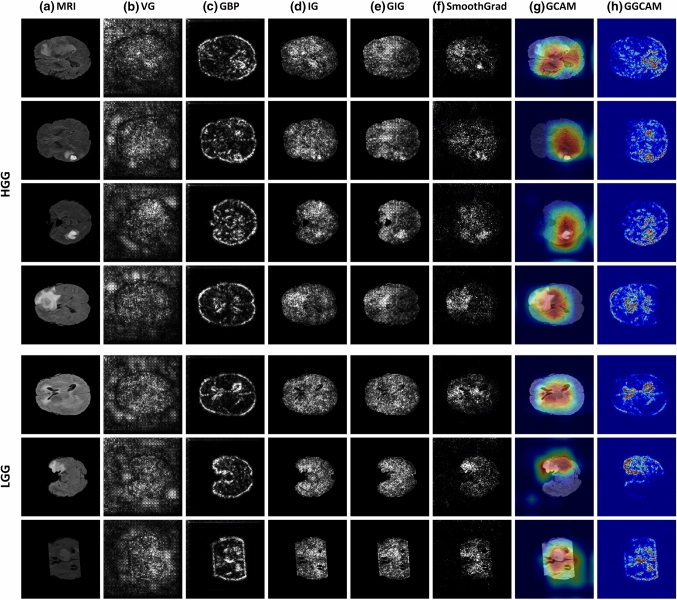
Comparing different XAI visualization methods for brain glioma classification. Sensitivity maps are presented for HGG cases in the first four rows, while for LGG cases in the last three rows. Left to right: original MRI image, Vanilla gradient, guided backpropagation, integrated gradients, guided integrated gradients, SmoothGrad, Grad-CAM, and guided Grad-CAM visualizations. Note that in (b, c, d, e, f), all contributing features are highlighted in white, while in (g, h), red regions correspond to a high score for the predicted class

To better understand the deep model’s prediction, we used the DL model to visualize various sensitivity maps using NeuroXAI as shown in Fig. 3. These 3D feature visualizations were generated from our model once the training is complete. Explanation maps by methods in (b-f) highlight all contributing features. In contrast, CAM heatmaps (g and h) highlight which regions of the input image are important for discriminating targeted classes.

Moreover, the visualization maps by pixel-space XAI methods, such as GBP, IG, and GIG, underlined fine-grained details in the input MRI image, but not being class-distinctive. In contrast, localization approaches like GCAM, are highly class-distinctive providing a smooth activation map. Notably, combining GBP with GCAM yielded better-localized visualizations with high resolution. SmoothGrad provided the best overall feature maps highlighting the main discriminative parts of the input FLAIR image so as to make the glioma grading. In contrast, VG provided noisy visualization maps compared with other methods due to the gradient saturation as reported in [41], making it less reliable for this application.

### Showcase II: application to segmentation

In this subsection, a feasible application of NeuroXAI is provided to interpret deep brain glioma sub-region segmentation using multimodal MRIs. Table 3 presents the comparison of the proposed segmentation model with the existing techniques on the BraTS validation dataset. Remarkably, our DL model has achieved the best dice score coefficient (DSC) of 84.10, 87.33, and 92 for the enhancing tumor, tumor core, and whole tumor regions, respectively.

**Table 3:** Comparison of our segmentation model and existing methods on the validation set

Model	Preprocessing	Method	DSC		
			ET*	TC*	WT*
DeepSeg (2D) [7]	FLAIR MRI, bias correction, data normalization, and transfer learning	2D U-Net	–	–	84.10
DeepSeg (3D) [38]	*Z*-score normalization and image cropping	3D U-Net	82.50	84.73	90.05
Ilhan et al. 2022 [42]	FLAIR MRI, tumor localization, and histogram equalization	U-net	–	–	0.88
nnU-Net [43]	Image cropping, data normalization, image resampling	U-Net	79.45	85.24	91.19
CASPIANET + + [44]	*Z*-score normalization	Attention U-Net	81.08	87.60	91.20
Our model	*Z*-score normalization, image cropping, on-the-fly data augmentation	ResNet-50	84.10	87.33	92

*ET, TC, and WT Enhancing tumor, tumor core, and whole tumor regions

**Fig. 4:**
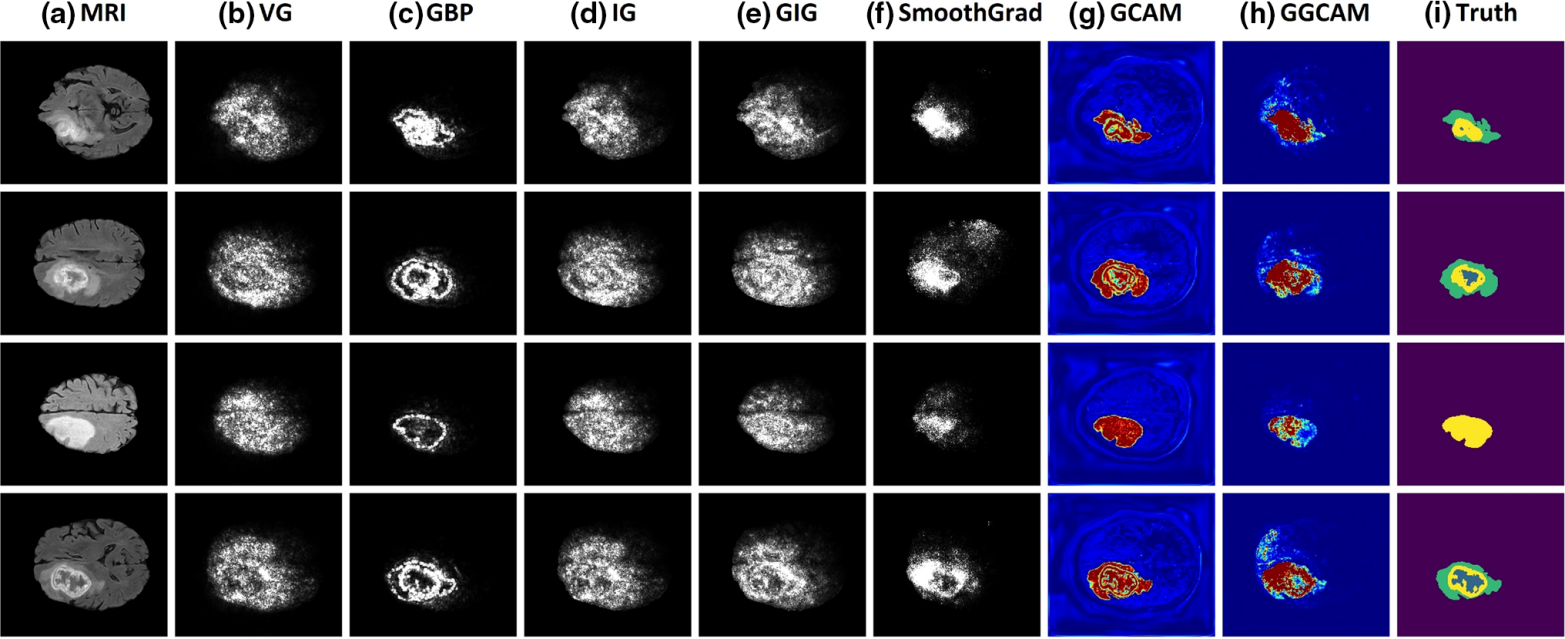
Comparing different XAI visualization methods for brain glioma segmentation. Left to right: original MRI image, Vanilla gradient, guided backpropagation, integrated gradients, guided integrated gradients, SmoothGrad, Grad-CAM, guided Grad-CAM, and the manual truth annotations. Note that in (**b, c, d, e, f**), all contributing features are highlighted in white, while in (**g, h**), red regions correspond to a high score for the tumor region

Figure 4 shows the qualitative results from different XAI methods for explaining our glioma segmentation network. It can be seen that the employed visualization methods generally clustered their attributions around the segmented brain tumor. In particular, GCAM, GGCAM, and SmoothGrad provided the least noisy visualization maps with the advantage of GCAM of being class discriminative. GBP generated high-resolution saliency maps in which the edges of the tumor sub-regions are highlighted instead of the tumor itself.

**Fig. 5:**
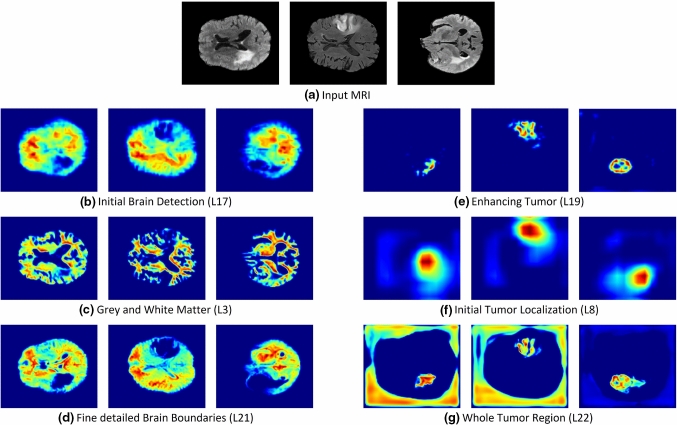
Visualization of the information flow in the segmentation CNN internal layers. The input MRI sequences are shown in **(a)**. **b**–**d** show implicit concepts for which no ground truth labels are available in addition to explicit concepts **e**–**g** with trained labels. L stands for convolutional layer

Besides, we analyzed each layer output toward the transparency of the black-box segmentation model. This experiment, explicitly network inspection, aims to clarify the flow of internal information in the neural network and whether this is in line with human-level concepts. For network inspection, GCAM was utilized since it allows visualizing activations in any layer of the deep network with respect to the network’s final output for a particular decision of interest. Figure 5 provides these explanation maps following the layers from the input MRI scans to the predicted segmentation map. These layer-wise importance maps show that the deep network follows a hierarchical nature similar to the human brain. For instance, layer 17 shows a neuron learning the initial brain boundaries, while the fine-grained brain localization was achieved later in layer 21. Similarly, the tumor was initially detected in layer 8, but the final precise segmentation was provided by the output layer.

Moreover, this deep neural network can learn some explicit concepts, which the CNN was not originally trained on, as well as implicit concepts from the underlying dataset. For instance, layer 22 in Fig. 5g seems to be learning the whole tumor region, as an explicit concept from the ground truth labeling data. Another example is shown in Fig. 5c for layer 3 learning the gray and white matter as an implicit concept which is not included in the training annotations.

## Discussion

DL has achieved the state-of-the-art in a wide range of medical tasks including medical image processing and analysis. By employing these AI advances in CADs, medical experts such as radiologists and surgeons become capable of detecting and diagnosing brain gliomas with great accuracy and shorter intervals. A deep neural network consists of numerous input, hidden, and output layers containing a large number of parameters (within millions). In applications increasingly vital to human healthcare, applying these models has been limited due to the lack of explainability.

NeuroXAI implements seven different gradient-based explanation methods, namely VG, GBP, IG, GIG, SmoothGrad, GCAM, and GGCAM, helping to make deep neural networks transparent. Each XAI method is unique and can be helpful in a different scenario with its inherent advantages and limitations. For example, VG is simple with the advantage of being supported by conventional machine learning frameworks such as TensorFlow [39] and PyTorch [45]. This makes VG applicable to any deep neural network without architectural modifications. On the other hand, the saliency maps generated by VG are noisy as well as they suffer from declining influences of features due to gradient saturation as reported in previous work [41]. GBP is efficient in terms of implementation; however, it is limited to CNN models with ReLU activations and does not provide class-distinctive visualization maps.

Recently, IG has become popular thanks to the ease of implementation, no requirement for instrumentation of the network, and fixed number of calls to the gradient. GIG is an enhancement to eliminate the false perturbations problem of IG, but a choice has to be made at every step at the path from baseline to input, and thus the direction of the path is not fixed. Although SmoothGrad can help improve visualizations of the overall true signal with the major drawback of being non-class discriminative, conversely, GCAM allows interpreting any convolutional layer of the CNN by highlighting the discriminative region and thus can help in understanding the internal functionality. To eliminate the lower-resolution heatmaps problem of GCAM, GGCAM was implemented as the combination of GBP and GCAM advantages.

These explanation methods and their application to two common applications for brain imaging analysis tasks, namely brain glioma grading and glioma localization, have been examined in detail. For both applications, high-resolution gradient-based saliency maps, including VG, GBP, IG, GIG, and SmoothGrad, highlight all contributing features, regardless of the selected class, as shown in Figs. 3 and 4. On the other hand, GCAM and GGCAM localize the most important regions for the network decision. This is consistent with findings in [27] showing that humans can better understand regions instead of pixels. Besides, network dissection, shown in Fig. 5, demonstrates that CNN follows a systematic approach for detecting the brain gliomas coherent with experts’ knowledge. First, the network learns the abstract features, such as the brain boundaries in Fig. 5a, and afterward identifies finely detailed tumor boundaries shown in Fig. 5c.

## Conclusions and outlook

This study presented a new explainability framework, named NeuroXAI, for assisting the interpretation of the behavior of DL networks using state-of-the-art visualization attention maps. NeuroXAI is post hoc and can therefore be applied to any deep neural models gaining insight into the behavior of these already trained models. Additionally, our two showcases have demonstrated the significance of incorporating XAI methods in medical image analysis tasks. NeuroXAI can also support the analysis of CNNs by providing an individual activation map for every internal filter. Moreover, our NeuroXAI results showed the importance of XAI for medical imaging tasks to understand DL models to accelerate their clinical acceptance by medical staff in the field.

Future work will be focused on the quantitative evaluation of XAI methods to assess the quality of the generated sensitivity maps and study their relationship with the DL accuracy metrics with additional experiments on multi-modal MRI-guided neurosurgery. Another main prospect of this research work is to investigate the possibility of extracting quantitative features from the explanation methods such as tumor volume and centroid.
